# Does stroke volume variation predict fluid responsiveness in children: A systematic review and meta-analysis

**DOI:** 10.1371/journal.pone.0177590

**Published:** 2017-05-12

**Authors:** Ling Yi, Zhongqiang Liu, Lina Qiao, Chaomin Wan, Dezhi Mu

**Affiliations:** 1 National Office for Maternal and Child Health Surveillance of China, West China Second University Hospital, Sichuan University, Chengdu, Sichuan, China; 2 Key Laboratory of Obstetric & Gynecologic and Pediatric Diseases and Birth Defects of Ministry of Education, Sichuan University, Chengdu, Sichuan, China; 3 Department of Pediatric Intensive Care Unit, West China Second University Hospital, Sichuan University, Chengdu, Sichuan, China; 4 Department of Pediatrics, West China Second University Hospital, Sichuan University, Chengdu, Sichuan, China; National Yang-Ming University, TAIWAN

## Abstract

**Objective:**

Stroke volume variation (SVV) is a reliable predictor of fluid responsiveness in adult patients. However, the predictive value of SVV is uncertain in pediatric patients. We performed the first systematic meta-analysis to evaluate the diagnostic value of SVV in predicting fluid responsiveness in children.

**Methods:**

PUBMED, EMBASE, and Cochrane Central Register of Controlled Trials were searched up to December 2016. Original studies assessing the diagnostic accuracy of SVV in predicting fluid responsiveness in children were considered to be eligible. A random-effects model was used to calculate pooled values of sensitivity, specificity and diagnostic odds ratio with 95% CI. The summary receiver operating characteristic curve was estimated and area under the curve was calculated. Quality of the studies was assessed with the QUADAS-2 tool.

**Results:**

Six studies with a total of 279 fluid boluses in 224 children were included. The analysis demonstrated a pooled sensitivity of 0.68 (95% CI,0.59–0.76), pooled specificity of 0.65 (95% CI, 0.57–0.73), pooled diagnostic odds ratio of 8.24 (95% CI, 2.58–26.30), and the summary area under the summary receiver operating characteristic curve of 0.81. However, significant inter-study heterogeneity was found (*p*<0.05, *I*^2^ = 61.3%), likely due to small sample size and diverse study characteristics.

**Conclusions:**

Current evidence suggests that SVV was of diagnostic value in predicting fluid responsiveness in children under mechanical ventilation. Given the high heterogeneity of published data, further studies are needed to confirm the diagnostic accuracy of SVV in predicting fluid responsiveness in pediatric patients.

## Introduction

Fluid therapy is the key intervention to improve tissue perfusion and oxygenation for critically ill and perioperative patients [1–3]. Both inadequate and excessive fluid infusion can lead to deleterious outcome for the patient [4, 5]. Thus, assessment of fluid responsiveness is essential to guide fluid resuscitation and optimize preload in perioperative medicine and critical care. Static measures of preload such as central venous pressure (CVP) and pulmonary artery wedge pressure (PAWP) were found to have poor predictive value in predicting fluid responsiveness [6–8]. Dynamic indices have emerged as promising predictors in recent years, and have been proven to predict fluid responsiveness far better than static measures [9–11]. Stroke volume variation (SVV) is one of the most widely used dynamic indices of fluid responsiveness in patients undergoing mechanical ventilation. SVV has been consistently shown to be reliable predictor of fluid responsiveness in adults. Zhang et al [12] reported that SVV was of diagnostic value for fluid responsiveness in adult patients ventilated with tidal volume greater than 8 ml/kg in operating room or intensive care unit. However, in the pediatric population, the predictive value of SVV remains relatively underexplored and the available results are controversial [13–15]. We hypothesised that SVV could be of predictive value in predicting fluid responsiveness in children. To test this hypothesis, we performed a systematic review and meta-analysis to summarize available evidence about the diagnostic accuracy of SVV in predicting fluid responsiveness in pediatric patients.

## Materials and methods

This systematic review and meta-analysis was conducted according to the Preferred Reporting Items for Systematic Reviews and Meta-Analyses statement (PRISMA) as shown in S1 File [16].

### Search strategy

Databases of PubMed, Embase, and Cochrane Central Register of Controlled Trials (CENTRAL) were searched for relevant publications with the following Medical Subject Headings and search terms: “fluid or volume or preload responsiveness”, “fluid or preload challenge”, “fluid therapy or management”, “stroke volume variation”. The initial search was conducted in August 2016 without language restrictions. The search was updated in December 2016 but did not identify any additional studies for inclusion. The search was conducted separately by two investigators (Z. L. and L.Y.).

### Study selection

The titles/abstracts for all articles from the search were reviewed, and full-text articles from potentially relevant abstracts were retrieved for assessment of eligibility. The bibliographies of all relevant articles were reviewed manually to identify additional relevant articles. Studies were included if the following criteria were fulfilled: (1) Studies investigated the ability of SVV to predict fluid responsiveness. (2) Studies involved pediatric subjects (age<18 years). (3) Data were available to calculate sensitivity, specificity. If raw data were not published but the study was otherwise eligible for inclusion, authors were contacted to obtain additional information.

### Data extraction

Data from all included studies were abstracted independently by two investigators (Z. L. and L.Y.) into a spreadsheet, with disagreement resolved by consensus. The following data were extracted from each included study: (1) characteristics of study (year of publication, study design, clinical setting), (2) characteristics of trial participants (number of patients, number of fluid bolus, age), (3) methods used to determine fluid responsiveness (monitoring device, fluid bolus type, tidal volume, definition of fluid responsiveness), (4) variables tested (cutoff value, sensitivity, specificity, the area under the receiver operating characteristic curve (AUROC)).

### Statistical analysis

A fluid bolus was employed as statistical unit as multiple fluid boluses were used in some patients. From the studies included, we extracted the numbers of fluid boluses with a true-positive, false-positive, true-negative, false-negative test result either directly or through recalculation. Sensitivity, specificity and diagnostic odds ratios (DOR) together with 95% CI for included studies were extracted or calculated based on the reconstructive contingency table. A random-effects model was used to calculate pooled values of sensitivity, specificity and DOR with 95% CI. A summary receiver operating characteristic (SROC) was constructed, and the AUROC was calculated. Statistical heterogeneity between studies was assessed using Cochran-*Q* and *I*^2^ tests [17]. Values of *p*<0.1 or *I*^2^>50% were considered to be statistically significant. Deek's Funnel Plot Asymmetry Test was applied to determine the presence of publication bias [18]. Meta-Disc (version 1.4) software was used for data analysis [19]. Subgroup analysis was performed according to the monitoring device the study used (non-invasive monitoring or invasive monitoring), and the type of patient population (postoperative patients or PICU patients).

## Results

### Study flow

**Fig 1:**
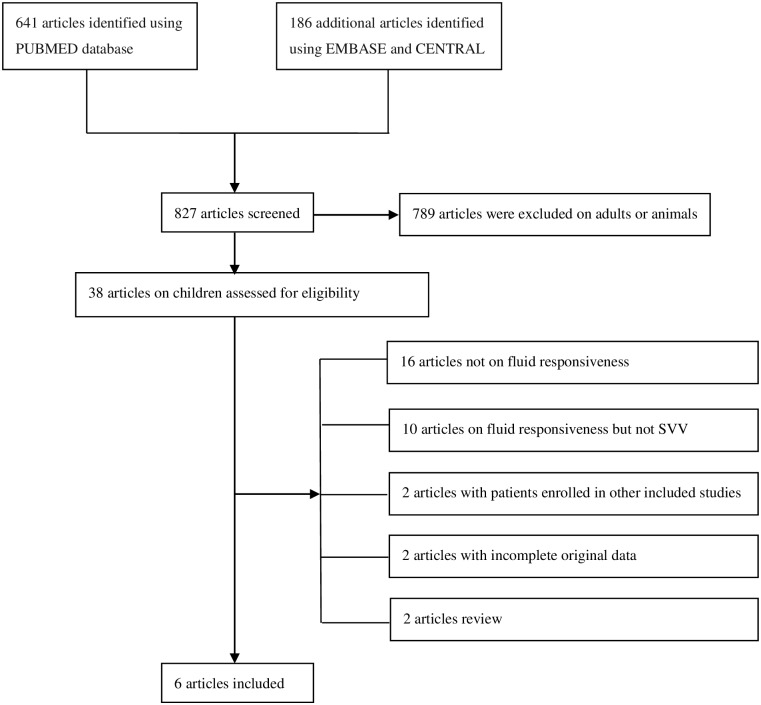
Flow chart of study selection and inclusion.

The flow chart of the study selection is illustrated in Fig 1. A total of 827 studies were identified after an initial search. Of them, 789 were excluded after title and abstract review. Full text review of the 38 potentially eligible articles was carried out and resulted in 32 articles being excluded. Reasons for exclusion are detailed in S2 File. Consequently, six studies were ultimately included in our analysis [13, 14, 20–23].

### Study characteristics

The characteristics of the individual studies are presented in Table 1. All of the studies were published from 2012 to 2015. Totally 279 fluid boluses were given in 224 children, as two studies used multiple fluid boluses in some patients [13, 20]. In the majority of studies, the patients were mechanically ventilated with tidal volumes of 10 ml/kg. Four studies used colloids as fluid bolus, one study used crystalloid, and one study did not report type of fluid given. Four studies were performed in the operating room during congenital heart surgery or craniosynostosis repair [14, 21–23], and two studies were performed in the PICU [13, 20]. SVV was tested by non-invasive measures such as NICOM (non-invasive cardiac output monitor) and USCOM (ultrasonic cardiac output monitor) in four studies, whereas two studies used invasive measures including PiCCO (pulse index continuous cardiac output) and Mostcare. In the majority of studies, fluid responsiveness was defined as change in stroke volume index (SVI) of at least 15%, except one study used a threshold of 10% [20]. The quality of the included studies were assessed by Quality Assessment of Diagnostic Accuracy Studies (QUADAS-2) available in Table 2 [24].

**Table 1:** Characteristics of included studies.

Study	Year	No. of patients	No. of fluid boluses	Patient population	Age	Tidal volume	Fluid bolus	Device	Definition of responders
Renner (14)	2012	26	26	Post cardiac surgery	14±2 months	10 ml/kg	10 ml/kg, colloids	PiCCO	ΔSVI_TOE_ ≥15%
McLean (20)	2014	13	26	PICU patients	2months-14years	-	10 ml/kg	USCOM	ΔSVI≥10%
Lee (21)	2014	26	26	Post cardiac surgery	6months-6years	10 ml/kg	10 ml/kg, colloids	NICOM	ΔSVI≥15%
Vergnaud (22)	2015	30	30	Post craniosynostosis repair	0-16years	7–8 ml/kg	20 ml/kg, colloids	NICOM	ΔSVI_TTE_ ≥15%
Lee (23)	2015	29	29	Post cardiac surgery	1–36 months	10 ml/kg	10 ml/kg, colloids	NICOM	ΔSVI_TOE_ ≥15%
Saxena (13)	2015	100	142	PICU patients	6–48 months	11.9±3.6 ml/kg	10 ml/kg, crystalloids	Mostcare	ΔSVI >15%

PICU: pediatric intensive care unit; PiCCO: pulse index continuous cardiac output; USCOM: ultrasonic cardiac output monitor; NICOM: non-invasive cardiac output monitor; SVI: stroke volume index; TOE: transoesophageal echocardiography; TTE: Transthoracic echocardiography.

**Table 2:** Quality assessment of included diagnostic accuracy studies using QUADAS-2.

Study	Risk of bias				Applicability concerns		
	Patient selection	Index test	Reference standard	Time and flow	Patient selection	Index test	Reference standard
Renner (14)	☺	☺	☺	☺	☺	☺	☺
McLean (20)	☺	☺	☺	☺	☺	☺	☺
Lee (21)	☺	☺	☺	**?**	☺	☺	☺
Vergnaud (22)	☹	☺	☺	**?**	☺	☺	☺
Lee (23)	☺	☺	☺	☺	☺	☺	☺
Saxena (13)	☺	☺	☺	☹	☺	☺	☺

☺ Low Risk; ☹ High Risk; **?** Unclear Risk

### Quantitative data synthesis

**Fig 2:**
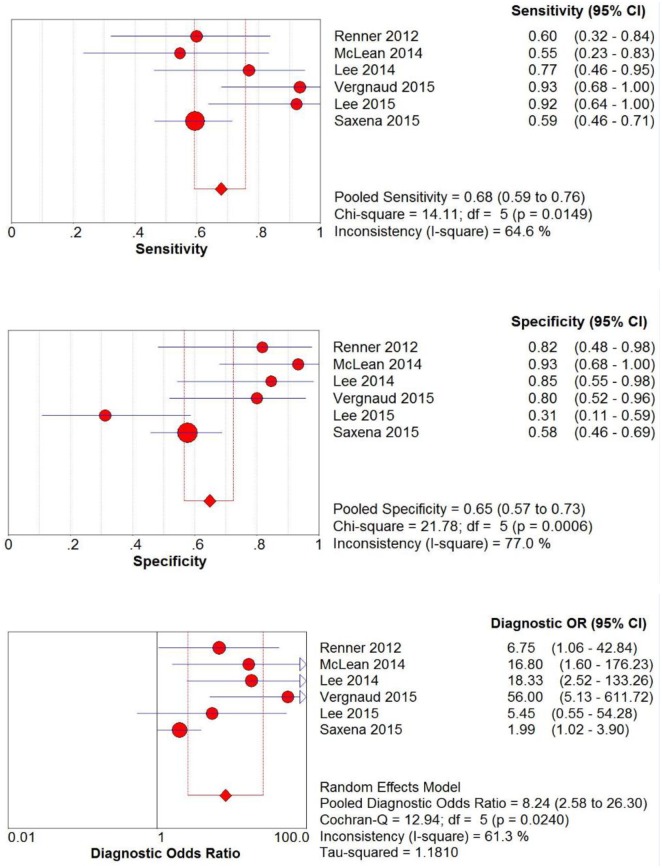
Sensitivity, specificity and diagnostic odds ratio of stroke volume variation in predicting fluid responsiveness in children assessed by forest plots. The point estimates of sensitivity, specificity and diagnostic odds ratio for each study are shown as solid circles.

**Fig 3:**
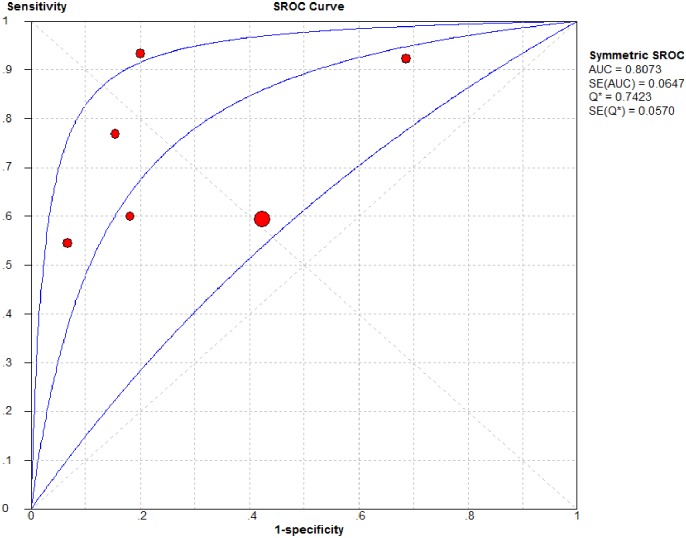
Summary receiver operating characteristic curve of stroke volume variation in predicting fluid responsiveness in children. Solid circles represent each study included in the meta-analysis.

Study data and individual diagnostic estimates are summarized in Table 3. Overall, 47% of the patients included in this review responded to fluid bolus. Cutoff values of SVV varied across studies, ranging from 10% to 22%. The AUROC of individual studies ranged from 0.51 to 0.89. There was significant heterogeneity between the included studies, the *I*^2^ for sensitivity, specificity and DOR were 64.6, 77.0 and 61.3, respectively. The pooled sensitivity, specificity and DOR from all six studies were 0.68 (95% CI, 0.59–0.76), 0.65 (95% CI, 0.57–0.73) and 8.24 (95% CI, 2.58–26.30) respectively (Fig 2). Fig 3 presents the receiver operator characteristic (ROC) scatter plot displaying the results of sensitivity and specificity for individual studies in the ROC space. A summary AUROC of 0.81 was obtained. There was evidence of small study effects (Deek's Funnel Plot Asymmetry Test, *p* = 0.04) (S1 Fig). In the subgroup analysis, summary AUROC for non-invasive monitoring studies and postoperative patients studies were 0.87 and 0.85 respectively (Table 4).

**Table 3:** Main results of individual studies.

Study	Reference	No. of responders	No. of non-responders	AUROC (95% CI)	Best threshold	Sensitivity (%)	Specificity (%)
Renner (14)	Renner	15	11	0.78 (0.61–0.97)	15%	0.60	0.81
McLean (20)	McLean	11	15	0.80(0.62–0.97)	16.5%	0.55	0.94
Lee (21)	Lee	13	13	0.89 (0.76–1.00)	10%	0.77	0.85
Vergnaud (22)	Vergnaud	15	15	0.81 (0.66–0.96)	10%	0.93	0.80
Lee (23)	Lee	13	16	0.51 (0.32–0.70)	17.6%	0.92	0.31
Saxena (13)	Saxena	64	78	0.53 (0.43–0.62)	22%	0.59	0.58

AUROC: area under the receiver operating characteristic curve.

**Table 4:** Main results of subgroup analysis.

Subgroup	No. of studies	No. of fluid boluses	Pooled sensitivity (95% CI)	Pooled specificity (95% CI)	DOR (95% CI)	*I*^2^ (%)	AUROC
All studies	6	279	0.68 (0.59–0.76)	0.65 (0.57–0.73)	8.24 (2.58–26.30)	61.3	0.81
Non-invasive monitoring subgroup	4	111	0.81 (0.67–0.90)	0.71 (0.58–0.82)	17.22 (5.63–52.69)	0.0	0.87
Postoperative patients subgroup	4	111	0.80 (0.68–0.90)	0.67 (0.53–0.79)	12.81 (4.49–36.51)	0.0	0.85

DOR: diagnostic odds ratio; AUROC: area under the receiver operating characteristic curve.

## Discussion

SVV has been demonstrated to be a reliable predictor of fluid responsiveness in adult patients [12]. However, few studies have addressed the reliability of SVV to predict fluid responsiveness in children. This is the first meta-analysis to examine the ability of SVV to predict the response to volume expansion in pediatric patients. We found six studies with a combined total of 224 patients. The results of meta-analysis showed a pooled sensitivity of 0.68, specificity of 0.65, and a summary AUROC of 0.81. A diagnostic tool with an AUROC of 0.81 is considered to have good diagnostic accuracy. Our results confirmed the diagnostic value of SVV in predicting fluid responsiveness in children under mechanical ventilation. However, high heterogeneity was found between studies, possibly due to small sample size and differences among monitoring devices, the fluid bolus was administered, and also to different patient characteristics.

In critically ill children, especially infants and neonates, adequate fluid resuscitation is a particular challenge for the pediatrician since fluid homeostasis is maintained in a narrow range and physiological compensation of both hypervolaemia and hypovolaemia is limited. Based on the Frank-Starling mechanism, the relationship between preload and stroke volume is curvilinear instead of linear. An increase in stroke volume will be induced by an increase in preload only if the ventricle operates on the ascending portion of the curve [25, 26]. Therefore, it is of great importance to assess each patient’s position on the Frank-Starling curve in order to optimize cardiac preload and avoid fluid overload. Static markers of cardiac preload were found to have poor predictive value of fluid responsiveness. To overcome the limitations of static indices, dynamic parameters have been introduced in clinical practice. Numerous dynamic parameters such as pulse pressure variation (PPV), systolic pressure variation (SPV), respiratory variation in aortic blood flow peak velocity (ΔVPeak) and Plethysmograph variability index (PVI) were investigated for prediction of fluid responsiveness in children [15]. There is no consensus on their predictive value in children except ΔVPeak [27].

In recent years, many investigations have been carried out to explore the SVV diagnostic accuracy in predicting fluid responsiveness. Numerous studies have demonstrated that SVV can predict fluid responsiveness reliably in adults [28–30]. A meta-analysis reported that SVV was an accurate predictor of fluid responsiveness in adult patients, with pooled sensitivity of 0.81, specificity of 0.80, and a summary AUROC of 0.93 [12]. The findings of our meta-analysis showed that SVV also have ability to predict fluid responsiveness in children, but the predictive value was not as encouraging as reported in adult patients. We speculated that it may be explained by the physiological differences between children and adult subjects, such as heart rate, chest wall compliance and vascular elasticity, all of which may influence SVV in a different way compared with adults.

Many studies demonstrated that tidal volume was an important factor that influences the predictive value of dynamic parameters. De Backer et al [31] reported that PPV can effectively predict fluid responsiveness in patients under mechanical ventilation only when tidal volume is at least 8 ml/kg. Suehiro et al [32] found that SVV was a reliable predictor of fluid responsiveness in patients undergoing one-lung ventilation provided with tidal volume of 8 ml/kg, while SVV was of no predictive value ventilated at 6 ml/kg. In our meta-analysis, three studies enrolled children mechanically ventilated with tidal volume of 10 ml/kg, the predictive value of SVV was positive in two studies. However, Lee et al [23] reported that SVV did not predict fluid responsiveness in pediatric patients during cardiac surgery ventilated with tidal volumes of 10 ml/kg. Furthermore, it was reported recently that the predictive value of dynamic parameters may be influenced by other settings of mechanical ventilation besides tidal volume in children. Kang et al [33] studied the influence of different peak inspiratory pressure (PIP) on SVV in pediatric cardiac surgery patients, and the result showed that SVV was affected by different levels of PIP in same patient and under same volume status. In consideration of the differences between the physiology in children and adult patients, further studies are needed to explore the optimal tidal volume and other parameters of mechanical ventilation for children.

The pulmonary artery catheter using the thermodilution technique has long been regarded as the gold standard approach for cardiac output monitoring. However, some studies showed that the usage of pulmonary artery catheter may cause complications associated with the insertion and may be related to increased risk in mortality [34, 35]. Alternative monitoring techniques currently available include esophageal Doppler technique (such as USCOM), transoesophageal echocardiography, thoracic electrical bioimpedance devices, pulse contour analysis (such as PiCCO and Mostcare), and bioreactance devices (such as NICOM) [34, 36, 37]. NICOM and USCOM were non-invasive monitoring measures which avoid the potential complications of pulmonary artery catheter insertion and maintenance. Several studies have shown that cardiac output measured by USCOM / NICOM had good accuracy compared with those obtained by the standard thermodilution-based techniques [38–41]. Monitoring devices used in the studies were diverse in our analysis, one study using USCOM, one study using PiCCO, one study using Mostcare, three studies using NICOM. Subgroup analysis results showed that SVV with non-invasive monitoring seems to have good value of prediction for fluid responsiveness in pediatric patients. Considering the limited number of studies using invasive monitoring involved in our analysis, more studies are needed to evaluate the predictive value of SVV monitoring with invasive devices.

Assessment and monitoring of fluid status is of great importance for both critically ill patients and perioperative patients to maintain hemodynamic stability. Zhang et al [12] reported that SVV was of diagnostic value for fluid responsiveness both in operating room and intensive care unit, with higher predictive value in the intensive care unit. In our subgroup analysis, the subgroup of postoperative patients appears to have higher value of prediction for fluid responsiveness. With respect to PICU patients involved in our analysis, only two sets of data were available, no definitive conclusion can be drawn in this subgroup of patients.

In our analysis, pediatric patients with a wide age range were enrolled in the studies, some included children younger than 5 years, and some included subjects in their late teens. The cardiovascular physiology changes remarkably from the neonatal period to one year of age, with minor changes thereafter [42, 43]. Myocardium is less responsive to preload and vulnerable to overfilling for newborns [44]. There are dramatic changes in arterial compliance and aortic characteristic impedance from newborns to teenager [45, 46]. Vergnaud et al [22] demonstrated that SVV reliably predicted fluid responsiveness only in children aged more than 3 years old, while AUROC was 0.57 in younger children. It is difficult to interpret the validity of summarized results from subjects of diverse age in our meta-analysis.

## Limitations

Several limitations should be considered when assessing the clinical relevance of our study results. Firstly, our analysis included only six studies with a relatively small sample size, among which the study implemented by Saxena et al was the largest study with more than 50% of the total combined 279 fluid boluses, thus the power and precision of the results were limited. Secondly, different monitoring devices were used in the studies. The quantity and type of fluid administered, and the cut-off values for defining fluid responsiveness varied obviously between the included studies. All these diverse study characteristics may influence the predictive value of SVV. Thirdly, some patients received vasoactive drugs, which might interfere with the cardiopulmonary interactions. Finally, we did not include studies without available data to calculate sensitivity and specificity, non-English studies, and unpublished studies, which may have increased the risk of reporting bias.

## Conclusions

This work is the first meta-analysis to evaluate the ability of SVV to predict the response to volume expansion in pediatric patients. Our results showed that SVV was of diagnostic value in predicting fluid responsiveness in children under mechanical ventilation. However, high heterogeneity was found between published studies, further studies are needed to confirm the diagnostic accuracy and utility of SVV in predicting fluid responsiveness in children.

## Supporting information

S1 File PRISMA checklist. (DOC)

S2 File Reasons for exclusion. (DOCX)

S1 Fig Deek's Funnel Plot Asymmetry Test for the assessment of potential publication bias. (TIFF)
