# Supplementary material for: Does stroke volume variation predict fluid responsiveness in children: A systematic review and meta-analysis
Source: PLoS One. 2017 May 12;12(5):e0177590. doi: 10.1371/journal.pone.0177590 (PMC5428964; doi:10.1371/journal.pone.0177590)
Supplement: S2 File — (DOCX) [file pone.0177590.s002.docx]

**Reasons for Exclusion**

**16 articles not on fluid responsiveness were excluded:**

**1. BMJ Open. 2014 Dec 8;4(12):e005360. doi: 10.1136/bmjopen-2014-005360.**

Effect of L-type calcium channel blocker (amlodipine) on myocardial iron deposition in patients with thalassaemia with moderate-to-severe myocardial iron deposition: protocol for a randomised, controlled trial.

Shakoor A1, Zahoor M1, Sadaf A1, Alvi N1, Fadoo Z1, Rizvi A1, Quadri F1, Tipoo FA2, Khurshid M3, Sajjad Z4, Colan S5, Hasan BS1.

Author information

1Department of Pediatrics and Child Health, Aga Khan University, Karachi, Sindh, Pakistan.

2Section of Cardiology, Department of Medicine, Aga Khan University, Karachi, Sindh, Pakistan.

3Section of Hematology and Oncology, Department of Medicine, Aga Khan University, Karachi, Sindh, Pakistan.

4Department of Radiology, Aga Khan University, Karachi, Sindh, Pakistan.

5Department of Cardiology, Boston Children's Hospital, Boston, Massachusetts, USA.

Abstract

INTRODUCTION:

Sideroblastic cardiomyopathy secondary to repeated blood transfusions is a feared complication in thalassaemia. Control of myocardial iron is thus becoming the cornerstone of thalassaemia management. Recent evidence suggests a role for L-type Ca(2+) channels in mediating iron uptake by the heart. Blocking the cellular iron uptake through these channels may add to the benefit of therapy to standard chelation in reducing myocardial iron. We aim to determine the efficacy of amlodipine (a calcium channel blocker) as an adjunct to standard aggressive chelation in retarding myocardial iron deposition in thalassaemics with or without cardiomyopathy.

OUTCOMES:

The primary outcome is to compare the efficacy of amlodipine+chelation (intervention) versus standard chelation (control) in retarding myocardial iron deposition. Secondary outcomes include the effect of amlodipine therapy on systolic and diastolic function, strain and strain rate and liver iron content.

METHODS AND ANALYSIS:

This is a single-centre, parallel-group, prospective randomised control trial. Twenty patients will be randomised in a 1:1 allocation ratio into the intervention and control arms. In addition to conventional echocardiography, MRI T2* values for assessment of cardiac and liver iron load will be obtained at baseline and at 6 and 12 months. Cardiac T2* will be reported as the geometric mean and per cent coefficient of variation, and an increase in cardiac T2* values from baseline will be used as an end point to compare the efficacy of therapy. A p Value of <0.05 will be considered significant.

STUDY SETTING:

Department of Pediatric and Child Health, Aga Khan University Hospital, Karachi, Pakistan.

ETHICS AND DISSEMINATION:

This study has been approved by the Ethics Review Committee and Clinical Trials Unit at The Aga Khan University with respect to scientific content and compliance with applicable research and human subjects regulations. Findings will be reported through scientific publications and research conferences and project summary papers for participants.

TRIAL REGISTRATION NUMBER:

ClinicalTrials.Gov. Registration no: NCT02065492.

Published by the BMJ Publishing Group Limited. For permission to use (where not already granted under a licence) please go to http://group.bmj.com/group/rights-licensing/permissions.

PMID: 25492271

**2. J Am Soc Echocardiogr. 2012 May;25(5):511-7. doi: 10.1016/j.echo.2012.01.011.** Epub 2012 Feb 16.

Right ventricular systolic strain is altered in children with sickle cell disease.

Blanc J1, Stos B, de Montalembert M, Bonnet D, Boudjemline Y.

Author information

1M3C-Necker, Paediatric Cardiology, Université Paris Descartes, Paris, France.

Abstract

BACKGROUND:

Several adult studies have shown that sickle cell disease is associated with cardiac abnormalities and premature death. The aim of this study was to use speckle-tracking strain, a relatively load independent parameter, to evaluate systolic left ventricular (LV) and right ventricular (RV) function in a pediatric sickle cell disease population.

METHODS:

Twenty-eight patients with sickle cell disease (mean age, 10.0 ± 3.6 years; mean body surface area, 1.14 ± 0.27 m(2)) and 29 controls matched for age and body surface area were compared. Cardiac output, LV dimension, wall thickness and circumferential strain, LV and RV longitudinal systolic strain, conventional and tissue Doppler parameters, and pulmonary pressure were assessed.

RESULTS:

LV cardiac output was significantly higher in patients, as were indexed LV systolic diameter, indexed LV mass, and E/E' septal ratio. Indexed LV diastolic diameter, wall thickness, LV shortening fraction, and global LV longitudinal and circumferential strains were similar in patients and controls. However, their global RV longitudinal strain was significantly lower, although tricuspid annular plane systolic excursion and color-coded tricuspid S-wave velocity were similar. Among patients, 21% had tricuspid regurgitation velocities > 2.5 m/sec, but none had tricuspid regurgitation velocities > 3 m/sec. Indexed LV diastolic dimension and systolic pulmonary artery pressure were significantly higher in patients whose hemoglobin was <80 g/L, but parameters of systolic and diastolic LV function were similar.

CONCLUSIONS:

In children with sickle cell disease, LV diastolic function is significantly altered, although LV systolic function, evaluated by global longitudinal strain, is normal. In addition, cardiac output is increased, and elevated tricuspid regurgitation velocity is common, whereas it is never found in controls. Most importantly, global RV longitudinal systolic strain is significantly altered.

Copyright © 2012 American Society of Echocardiography. Published by Mosby, Inc. All rights reserved.

PMID: 22341367

**3. Pediatr Radiol. 2011 Aug;41(8):1000-7. doi: 10.1007/s00247-011-2033-3. Epub 2011 Mar 25.**

Repeatability of cardiac-MRI-measured right ventricular size and function in congenital heart disease.

Walsh R1, Salem Y, Shah A, Lai WW, Nielsen JC.

Author information

1Division of Pediatric Cardiology, Mount Sinai School of Medicine, New York, NY, USA.

Abstract

BACKGROUND:

The measurement error for right ventricular (RV) size and function assessed by cardiac MRI (CMRI) in congenital heart disease has not been fully characterized. As CMRI parameters are being increasingly utilized to make clinical decisions, defining error in the clinical setting is critical.

OBJECTIVE:

This investigation examines the repeatability of CMRI for RV size and function.

MATERIALS AND METHODS:

Forty consecutive people with congenital heart disease involving the RV were retrospectively identified. Contouring of RV volumes was performed by two expert CMRI clinicians. The coefficient of variability and repeatability coefficients were calculated. Repeatability coefficients were multiplied by the mean value for each group studied to define a threshold beyond which measurement error was unlikely to be responsible.

RESULTS:

The variability for indexed RV end-diastolic volume = 3.2% and 3.3% for intra- and interobserver comparisons, respectively. The repeatability coefficients were 13.2% and 14.9% for intra- and interobserver comparisons, which yielded threshold values of 15.1 ml/m^2 and 20.2 ml/m^2, respectively. For RV ejection fraction (EF), the repeatability coefficients for intra- and interobserver comparisons were 5.0% and 6.0%, which resulted in threshold values of 2.6 EF% and 3.0 EF%.

CONCLUSION:

The threshold values generated can be used during serial assessment of RV size and function.

PMID: 21437608

**4. J Am Coll Cardiol. 2011 Mar 1;57(9):1100-7. doi: 10.1016/j.jacc.2010.09.063.**

Isovolumic acceleration at rest and during exercise in children normal values for the left ventricle and first noninvasive demonstration of exercise-induced force-frequency relationships.

Roche SL1, Vogel M, Pitkänen O, Grant B, Slorach C, Fackoury C, Stephens D, Smallhorn J, Benson LN, Kantor PF, Redington AN.

Author information

1The Labatt Family Heart Centre, The Hospital for Sick Children, University of Toronto, Toronto, Ontario, Canada.

Abstract

OBJECTIVES:

This study aimed to determine the normal variation of left ventricular (LV) isovolumic acceleration (IVA) in healthy children and to assess the feasibility of an entirely noninvasive method for demonstration of the LV force-frequency relationship (FFR).

BACKGROUND:

Pediatric cardiologists continue to seek noninvasive, load-independent indexes for the assessment of LV contractility and myocardial reserve.

METHODS:

Resting LV IVA was measured by echocardiogram in 236 healthy children and compared with their clinical characteristics. Further measurements were made in 51 children at incremental heart rates during semi-recumbent exercise. For these, FFRs were constructed by plotting LV IVA against heart rate. To assess potential clinical applications, pilot FFR data were collected from 16 children previously treated with anthracyclines.

RESULTS:

In healthy children, median resting LV IVA was 1.2 m/s(2), interquartile range 0.9 to 1.6 m/s(2). Resting LV IVA was unaffected by age, sex, weight, height, and body surface area but associated with baseline heart rate (r = 0.18, p = 0.0006). Noninvasive evaluation of the LV FFR was possible in 98% of subjects. Positive FFRs were confirmed in all the healthy children. By comparison, several of the children with anthracycline exposure demonstrated flattened force-frequency curves that were largely independent of resting LV ejection fraction and suggest reduced contractile reserve.

CONCLUSIONS:

In children over 7 years, it is possible to demonstrate the LV FFR by interval measurement of IVA during exercise. The availability of pediatric normal values for both this relation and resting LV IVA might facilitate future investigation of LV contractility and myocardial contractile reserve during childhood.

Copyright © 2011 American College of Cardiology Foundation. Published by Elsevier Inc. All rights reserved.

Comment in

How the (pediatric) heart works when it contracts application of left ventricular "isovolumic acceleration" as a load-independent index of contractility. [J Am Coll Cardiol. 2011]

PMID: 21349402

**5. Int J Cardiol. 2011 Mar 3;147(2):214-8. doi: 10.1016/j.ijcard.2009.08.024. Epub 2009 Sep 9.**

Safety and observer variability of cardiac magnetic resonance imaging combined with low-dose dobutamine stress-testing in patients with complex congenital heart disease.

Robbers-Visser D1, Luijnenburg SE, van den Berg J, Roos-Hesselink JW, Strengers JL, Kapusta L, Moelker A, Helbing WA.

Author information

1Department of Paediatrics, Division of Cardiology, Erasmus MC - Sophia Children's Hospital, Rotterdam, The Netherlands.

Abstract

BACKGROUND:

In patients with complex congenital heart disease (CHD) abnormal ventricular stress responses have been reported with dobutamine stress cardiovascular magnetic resonance (DCMR). These abnormal stress responses are potential indicators of long-term outcome. However, safety and reproducibility of this technique has not been reported in a larger study. The aim of this study was to report our experiences regarding safety and intra-observer and inter-observer variability of low-dose DCMR in complex CHD.

METHODS:

In 91 patients, 110 low-dose DCMR studies were performed with acquisition of a short axis set at rest, and during dobutamine administration (7.5 μg/kg/min maximum). We assessed biventricular end-diastolic volumes, end-systolic volumes, stroke volumes, ejection fraction and ventricular mass. Intra- and inter-observer variability for all variables was assessed by calculating the coefficient of variation (%), i.e. the standard deviation of the difference divided by the mean of 2 measurements multiplied by 100%.

RESULTS:

In 3 patients minor side effects occurred (vertigo, headache, and bigeminy). Ten patients experienced an increase in heart rate of >150% from baseline, although well tolerated. For all variables, intra-observer variability was <10% at rest and during stress. At rest, inter-observer variability was 10.5% maximal. With stress-testing, only the variability of biventricular end-systolic volumes (ESV) exceeded 10%.

CONCLUSIONS:

In patients with complex CHD low-dose DCMR is feasible, and safe. Intra-observer variability is low for rest and stress measurements. Inter-observer variability of biventricular ESV is high with stress-testing. Whether this limits the potential usefulness of DCMR for risk assessment during follow-up has to be assessed.

Copyright © 2009 Elsevier Ireland Ltd. All rights reserved.

PMID: 19740557

**6. Am J Cardiol. 2007 Apr 1;99(7):974-7. Epub 2007 Feb 16.**

Acute cardiac functional and morphological changes after Anthracycline infusions in children.

Ganame J1, Claus P, Eyskens B, Uyttebroeck A, Renard M, D'hooge J, Gewillig M, Bijnens B, Sutherland GR, Mertens L.

Author information

1Pediatric Cardiology Department, University Hospitals Leuven, Leuven, Belgium. javier.ganame@uz.kuleuven.ac.be

Abstract

The aim of this study was to describe the acute effects of anthracyclines on left ventricular systolic and diastolic function using different echocardiographic modalities. Thirteen children scheduled to receive anthracyclines were prospectively studied. They underwent complete 2-dimensional and Doppler echocardiographic evaluations, including tissue Doppler imaging, before the first dose and<2 hours after each of the first 3 doses of anthracyclines (dose range 30 to 75 mg/m2). After the first dose, increased end-diastolic wall thickness, decreased wall thickening, and a prolonged myocardial performance index were noted. Parameters of diastolic function changed significantly, with a lower mitral E wave, a decreased E/A ratio, and prolonged isovolumic relaxation time. Also, reduced longitudinal early diastolic myocardial velocity and myocardial velocity acceleration during isovolumic contraction as well as reduced peak longitudinal and radial systolic strain rate and strain were noted. All these parameters remained significantly lower after subsequent doses. After the second dose, significant changes in the shortening fraction and the ejection fraction compared with baseline became apparent. After the third dose, further deterioration in radial peak systolic strain was seen. In conclusion, low to moderate doses of anthracyclines acutely induce cardiac diastolic and systolic dysfunction.

PMID: 17398195

**7. J Am Soc Echocardiogr. 2003 Mar;16(3):214-20.**

Left ventricular remodeling, mechanics, and tissue characterization in congenital aortic stenosis.

Pacileo G1, Calabrò P, Limongelli G, Russo MG, Pisacane C, Sarubbi B, Calabrò R.

Author information

1Pediatric Cardiology Division, 2 Degrees University, Monaldi Hospital. gpacile@tin.it

Abstract

BACKGROUND:

As the response of the myocardium to pressure overload is age-dependent, this study was designed to examine left ventricular (LV) remodeling, mechanics, and tissue characterization in children with moderate congenital aortic stenosis.

METHODS:

We studied by echocardiography Doppler 22 patients (mean age 12.4 +/- 5.6 years) with peak and mean transvalvular gradient of 63 +/- 6 and 32 +/- 4 mm Hg, respectively. In addition, 30 age- and body surface area-matched participants with structurally normal hearts were used as a control group. Sex- and age-specific cut-off levels for LV mass/height(2.7) and relative wall thickness were defined to assess LV geometry. As a load-independent index of myocardial contractility, the relation between the rate-corrected velocity of circumferential fiber shortening both at endocardium and midwall, and meridional end-systolic stress was assessed. In addition, LV diastolic function was also evaluated by the mitral flow indexes. Finally, ultrasonic tissue characterization of the LV myocardium was performed by calculating the magnitude of cyclic variation, which reflects the intramural contractile function, and the averaged myocardial intensity of integrated backscatter, which is directly related to the myocardium collagen content.

RESULTS:

The endocardial velocity of circumferential fiber shortening endocardium and meridional end-systolic stress relationship was within the normal range (mean +/- 2SD) in 18 of 22 patients (81.8%), and midwall velocity of circumferential fiber shortening at endocardium and meridional end-systolic stress was normal in all 22 patients. No mitral flow index of LV diastolic function was significantly different between aortic stenosis group and normal participants. In our study population, 16 of 22 patients (72.7%) showed normal LV geometry, 3 (13.6%) had a pattern of concentric remodeling, and 3 (13.6%) concentric hypertrophy. LV hypertrophy was not marked (left ventricular mass index [LVMI] < 51 g/m(2.7)) in any patient. Finally, compared with control participants our study population showed, both at interventricular septum and posterior wall, comparable values of cyclic variation integrated backscatter, but significantly higher values of averaged myocardial integrated backscatter intensity (P <.01).

CONCLUSIONS:

In children with moderate congenital aortic stenosis, the total amount of myocardial collagen was increased despite normal LV myocardial contractility and diastolic function. Furthermore, LV remodeling was abnormal in only about a quarter of our patients and none had more than mild hypertrophy. Although the majority of these patients do not have markers now recognized to predict higher risk of cardiovascular events, the long-term significance of myocardial fibrosis and its response to treatment remain to be investigated.

PMID: 12618728

**8. Eur J Appl Physiol. 2001 Jun;84(6):547-56.**

Oxygenation trends in vastus lateralis muscle during incremental and intense anaerobic cycle exercise in young men and women.

Bhambhani Y1, Maikala R, Esmail S.

Author information

1Faculty of Rehabilitation Medicine, University of Alberta, Edmonton, Canada. yagesh.bhambhani@ualberta.ca

Abstract

The purposes of this study were to compare the acute cardiorespiratory responses and muscle oxygenation trends during incremental cycle exercise to exhaustion with those observed during 30 s and 45 s Wingate tests in healthy men and women, and to examine the relationships between selected variables among these tests. Seventeen healthy junior badminton players, nine men [mean age, height, body mass and maximal oxygen uptake (VO2max) were 15.8 (SD 0.8) years, 1.73 (SD 0.08) m, 65.6 (SD 6.3) kg and 50.6 (SD 6.9) ml x kg(-1) x min(-1) respectively] and eight women [mean age, height, body mass and VO2max were 16.6 (SD 1.0) years, 1.65 (SD 0.03) m, 62.7 (SD 4.5) kg and 42.0 (SD 5.0) ml x kg(-1) x min(-1) respectively] completed a stepwise incremental exercise test to voluntary exhaustion and two Wingate tests lasting 30 s and 45 s in three separate sessions in random order. Cardiorespiratory responses were monitored breath-by-breath using a metabolic cart interfaced with an electrocardiogram. Tissue absorbancy trends were continuously recorded from the right vastus lateralis muscle using dual wavelength near infrared spectroscopy. Oxygen uptake and heart rate were significantly higher during the incremental test when compared to the two Wingate tests in the men and women. However, the oxygen pulse (oxygen utilization per heart beat, i.e., the product of stroke volume and arterio-venous oxygen difference) was not significantly different among the three tests in both sexes. The minimal tissue absorbancy, an index of muscle deoxygenation, was also not significantly different among the three tests in both sexes. Significant relationships were observed for the oxygen uptake (r2=0.72) and oxygen pulse (r2=0.60) between the incremental and 45 s Wingate tests in the sample for both sexes combined. The minimal tissue absorbancy, however, was not significantly related between the two tests. It was concluded that the significantly higher oxygen uptake during the incremental test was due to the higher heart rate because: firstly, oxygen pulse was not significantly different among the three tests, and secondly, peripheral factors, as indicated by the changes in muscle oxygenation, were not significantly different among the three test conditions. Although the peak values of the oxygen pulse during the incremental and 45 s Wingate tests were significantly correlated, the common variance of the minimal tissue absorbancy measurements between these two tests was quite low, suggesting considerable variation in the peripheral contribution during these two tests.

PMID: 11482550

**9. Early Hum Dev. 1997 Apr 25;48(1-2):1-9.**

Changes in right ventricular volume in early human neonates.

Tamura M1, Harada K, Ito T, Takahashi Y, Ishida A, Takada G.

Author information

1Department of Pediatrics, Akita University School of Medicine, Japan.

Abstract

To evaluate changes in the right ventricular volume in early human neonates, twenty fullterm infants were examined at 2, 24 and 120 h of age by two-dimensional echocardiography. End-diastolic and end-systolic right ventricular volumes (RVEDV and RVESV, respectively) were calculated with a computer system based on the bi-plane Simpson's rule using the apical four chamber and parasternal short axis views. Then right ventricular stroke volume (RVSV), ejection fraction (RVEF), and the mean normalized systolic ejection rate were obtained. The inner diameter of the ductus arteriosus was also measured simultaneously. RVEDV increased significantly by 24 h of age, but remained constant thereafter. RVESV remained virtually unchanged from 2 to 120 h, resulting in a significant increase (36%) of RVSV at 24 h compared with that at 2 h. The mean normalized systolic ejection rate remained unchanged. There was a good correlation between RVEDV and RVSV (r = 0.83). All ductus arteriosus except three narrow ones was closed by 24 h of age. In conclusion, at 24 h of age, the significantly increased RVEDV was closely related to the increased RVSV, which might be induced by increased volume load to the right ventricle because of the closure of the ductus arteriosus.

PMID: 9131302

**10. Acta Paediatr. 1995 Jul;84(7):761-4.**

Continuous haemodynamic monitoring in children: use of transoesophageal Doppler.

Murdoch IA1, Marsh MJ, Tibby SM, McLuckie A.

Author information

1Department of Paediatric Intensive Care, Guy's Hospital, London, UK.

Abstract

A wide range of invasive and non-invasive techniques for monitoring the haemodynamic condition of critically ill patients is now available. A general reluctance on the part of paediatric intensive care specialists to use pulmonary artery thermodilution catheters and the need for constant realignment of hand-held Doppler probes has necessitated the search for a technique which is relatively non-invasive and provides continuous information on the haemodynamic condition of critically ill paediatric patients. We sought to establish if transoesophageal Doppler fulfilled these criteria. Eleven children who had recently undergone cardiac surgery were studied. Median age was 39 months and weight 14.9 kg. Five simultaneous pairs of measurements of cardiac index (CI: thermodilution) and minute distance (MD: transoesophageal Doppler) were made, as a baseline, when each child was haemodynamically stable. Following a fluid challenge, five repeat pairs of measurements were made. The mean percentage changes for CI and MD were 16.4% (range 5.3-44%) and 16.6% (3.4-47.7%), respectively. The average coefficients of variation for measurements of CI and MD were 3.5% and 2.9%, respectively. The mean difference in percentage change between CI and MD was -0.5% (95% confidence interval for the bias -4% to 3%; limits of agreement -10.7 to +9.7%). Our study indicates that transoesophageal Doppler is reproducible, easy to use and provides clinically acceptable information when following changes in CI in haemodynamically stable paediatric patients.

PMID: 7549293

**11. Pediatr Pulmonol. 1994 Jun;17(6):370-7.**

Effective pulmonary blood flow in children with acute asthma attack requiring hospitalization.

Yiallouros PK1, Milner AD.

Author information

1Division of Paediatrics, United Medical School of Guy's Hospital, University of London, England.

Abstract

In children with acute obstructive lung disease gas exchange is affected by ventilation-perfusion mismatch and the degree of bronchoconstriction. Standard lung function measurements do not reflect the impairment in gas exchange. Alternatively, the effective pulmonary blood flow (EPBF), that is, the proportion of the cardiac output that is supplying well-ventilated lung units, can give accurate and noninvasive estimates of ventilation-perfusion mismatch. We measured EPBF with the argon freon-22 rebreathing technique in children with acute severe asthma to assess their response to nebulized salbutamol and to determine whether induced changes in the EPBF could be predicted from baseline measurements. Twenty-four children admitted with an acute asthma attack had spirometry and triplicate EPBF measurements before and after nebulized salbutamol. Eighteen patients had repeated tests 50 days later when fully recovered; 4 patients were taking methylxanthines on at least one occasion. The mean forced expiratory volume in 1 sec (FEV1) rose from 55% of predicted to 66% after salbutamol and to 83% with recovery. The mean coefficients of variation for EPBF measurements on the three test occasions were 11.3%, 8.2%, and 9%. Except in children on methylxanthines, the EPBF values were reduced during the acute asthma attack (median, 2.53 L/min/m2; range, 1.99-3.60 L/min/m2) compared with paired values obtained after recovery (median, 2.89 L/min/m2; range, 2.28-4.04 L/min/m2) (P = 0.009). Salbutamol caused a highly significant increase in EPBF from 2.88 L/min/m2 (range, 1.86-3.80) before treatment to 3.34 L/min/m2 (range, 2.26-4.65) immediately afterwards (P = 0.0003).(ABSTRACT TRUNCATED AT 250 WORDS)

PMID: 8090607

**12. Heart Vessels. 1994;9(4):210-7.**

Time course of oxygen uptake and heart rate during Bruce treadmill test in patients following surgery for tetralogy of Fallot.

Tatara K1, Matsuoka S, Kubo M, Ushiroguchi Y, Kuroda Y.

Author information

1Department of Pediatrics, School of Medicine, University of Tokushima, Japan.

Abstract

Exercise performance was examined in 14 patients with a prior history of surgical repair for tetralogy of Fallot. Using a Bruce protocol, we evaluated the time course of oxygen uptake and heart rate in these patients and compared them with a standard curve obtained from 63 sex- and age-matched healthy children. Five of the 14 patients had unusual findings. The heart rate increased rapidly in four of them, while the oxygen uptake increased gradually (95% confidence lower limit). It is hypothesized that a reduction in stroke volume during exercise was responsible for these abnormal findings. The remaining unusual finding was in a patient who was known to have myocardial fibrosis and vacuolization. In this particular case, the time course of oxygen uptake and heart rate were both below the 95% coefficient of variation from the beginning to the end of exercise. In patients who have undergone surgical repair for tetralogy of Fallot, the analysis of oxygen uptake time course and the heart rate may provide valuable information for the long-term follow-up.

PMID: 7961299

**13. Br Heart J. 1988 Oct;60(4):316-23.**

Longitudinal study of ventricular function after the Mustard operation for transposition of the great arteries: a long term follow up.

Wong KY1, Venables AW, Kelly MJ, Kalff V.

Author information

1Department of Cardiology, Royal Childrens' Hospital, Melbourne, Australia.

Abstract

An earlier study of 25 patients who were investigated by radionuclide angiography after a Mustard procedure showed that they had had evidence of right and left ventricular dysfunction at rest and with exercise. Twenty one (mean age 17.0 years (range 13.7-20.6) 11 female patients) of the original 25 patients were followed up a mean of 4.3 years later (mean 14.6 years (range 12.5-16.0) after the procedure). The group means for resting right and left ventricular ejection fraction and exercise response were not significantly different from those reported five years before. Individual changes in values were within the normal variation seen in serial studies. This long term longitudinal follow up of patients after the Mustard operation showed that although some patients still had right and left ventricular dysfunction, resting ventricular function and exercise response remained stable over a five year period. This preservation of cardiac function may contribute to the long term survival of patients after the Mustard procedure.

PMID: 3190960

**14. Arch Mal Coeur Vaiss. 1985 May;78(5):771-6.**

[Radioisotopes for the study of right and left ventricular function in surgically treated Fallot's tetralogy].

[Article in French]

Brunotte F, Marçon F, Cloez JL, Laurens MH, Itty C, Robert J, Pernot C.

Abstract

Radio-nuclide angiography after surgical correction of tetralogy of Fallot (TOF) allows measurement of the ejection fraction, especially of the right ventricle, under basal conditions and on effort, parameters which are difficult to measure by other non-invasive methods. Twenty-two children with a mean age of 12.6 +/- 6.9 years who had undergone complete correction of TOF at a mean age of 6.7 +/- 3.8 years underwent Technetium 99m blood pool studies at equilibrium. The results were compared with those of a control group of children with a mean age of 10.2 +/- 3.3 years. No first passage studies were performed because the frequency of postoperative pulmonary regurgitation does not allow quantification of the shunt due to a possible residual ventricular septal defect. No significant difference was found between the patients and control subjects at rest: LV ejection fraction (66.7 +/- 11 p. 100 vs 63 +/- 7.7 p. 100), RV ejection fraction (50.3 +/- 7.2 p. 100 vs 54 +/- 14.8 p. 100). The response to effort of the right and left ventricles depends on the type of exercise. The LV ejection fraction increased normally whilst the RV ejection fraction showed a lot of individual variation. Equilibrium radionuclide angiography was also used to calculate the ratio of right to left end diastolic ventricular volumes. This ratio indicates the degree of RV diastolic overload when LV diastolic volumes are normal, which was the case in our series. The study group showed a significant increase in this ratio compared to control subjects (1.94 +/- 0.65 vs 1.2 +/- 0.23). Exercise radionuclide imagery should provide more accurate assessments of the surgical results and prognosis of these children providing standardised protocols are used. These investigations are best performed in patients over 15 years old, so as to avoid the technical difficulties related to small size. Resting studies are possible at all ages.

PMID: 3925921

**15. Anaesthesia. 1983 Mar;38(3):243-9.**

Labetalol in tetanus. The treatment of sympathetic nervous system overactivity.

Wesley AG, Hariparsad D, Pather M, Rocke DA.

Abstract

The cardiovascular instability in some cases of severe tetanus is due to increased circulating catecholamines. In 15 patients with this complication of tetanus, labetalol, a drug with alpha- and beta-peripheral adrenergic blocking properties, was used in management. The drug was administered orally, by i.v. bolus or by continuous infusion. Wide variation in dosage was needed both from case to case and in the same patient during the course of his disease. In most cases the pulse and blood pressure were reduced by labetalol, although their variability was not much improved. The effect on the cardiac output and systemic vascular resistance ranged from modest reduction in both, to marked effect on cardiac output without any change in systemic vascular resistance. The alpha-adrenergic blockage of labetalol is known to be less powerful than the beta-blocking effect, and this property could be a disadvantage in the management of sympathetic overactivity in tetanus.

PMID: 6837902

**16. Am Heart J. 1982 Jul;104(1):85-91.**

Cardiac response to exercise in patients with chronic aortic regurgitation.

Peter CA, Jones RH.

Abstract

Rest and exercise measurements of left ventricular (LV) ejection fraction (EF) and volumes were obtained by radionuclide angiocardiography (RNV) in 30 patients with severe aortic regurgitation (AR). The ratio of peak systolic pressure to end-systolic volume was used as an index of contractility. Volumetric cardiac output (CO) averaged 11.7 +/- 3.8 L/min at rest and 18.4 +/- 5.6 L/min during exercise. Much individual variation occurred in LVEF and end-diastolic volume (EDV) responses to exercise, and there was no consistent change in these measurements. Resting hemodynamic parameters and clinical history correlated poorly with changes observed during exercise. An increase in heart rate was one mechanism used by all 30 patients to increase CO during exercise. An inverse relationship was defined between the change in myocardial contractility and the change in EDV during exercise. Patients with the greatest increase in contractility during exercise showed the greatest decrease in EDV. Less use of an exercise increase in contractility was associated with an exercise increase in EDV to meet the demand for greater CO. Therefore, exercise measurements of LV function provide unique information regarding the degree of impairment of the LV myocardium in these patients with chronic AR.

PMID: 7090988

**11 articles on fluid responsiveness but not stroke volume variation were excluded:**

**1. Indian J Anaesth. 2016 Feb;60(2):121-6. doi: 10.4103/0019-5049.176285.**

Respiratory variation in aortic flow peak velocity and inferior vena cava distensibility as indices of fluid responsiveness in anaesthetised and mechanically ventilated children.

Achar SK1, Sagar MS1, Shetty R2, Kini G1, Samanth J3, Nayak C3, Madhu V3, Shetty T3.

Author information

Abstract

BACKGROUND AND AIMS:

Dynamic parameters such as the respiratory variation in aortic flow peak velocity (ΔVpeak) and inferior vena cava distensibility index (dIVC) are accurate indices of fluid responsiveness in adults. Little is known about their utility in children. We studied the ability of these indices to predict fluid responsiveness in anaesthetised and mechanically ventilated children.

METHODS:

This prospective study was conducted in 42 children aged between one to 14 years scheduled for elective surgery under general endotracheal anaesthesia. Mechanical ventilation was initiated with a tidal volume of 10 ml/kg. ΔVpeak, dIVC and stroke volume index (SVI) were measured before and after volume expansion (VE) with 10 ml/kg of crystalloid using transthoracic echocardiography. Patients were considered to be responders (R) and non-responders (NR) when SVI increased to either ≥15% or <15% after VE. ΔVpeak and dIVC were analysed between R and NR.

RESULTS:

The best cut-off value for ΔVpeak as defined by the receiver operator characteristics (ROC) curve analysis was 12.2%, for which sensitivity, specificity, positive predictive value and negative predictive value were 100%, 94%, 96% and 100%, respectively, the area under the curve was 0.975. The best cut-off value for dIVC as defined by the ROC curve analysis was 23.5%, for which sensitivity, specificity, positive predictive value and negative predictive value were 91%, 89%, 91% and 89%, respectively, the area under the curve was 0.95.

CONCLUSION:

ΔVpeak and dIVC are reliable indices of fluid responsiveness in children.

KEYWORDS:

Central venous pressure; echocardiography; paediatric anaesthesia; stroke volume; vena cava; volume expansion

PMID: 27013751

**2. Br J Anaesth. 2015 Jul;115(1):45-52. doi: 10.1093/bja/aev118. Epub 2015 May 19.**

A randomised feasibility study to assess a novel strategy to rationalise fluid in patients after cardiac surgery.

Parke RL1, McGuinness SP2, Gilder E3, McCarthy LW3, Cowdrey KA3.

Author information

1Cardiothoracic and Vascular Intensive Care Unit, Auckland City Hospital, Auckland, New Zealand Australian and New Zealand Intensive Care Research Centre, Department of Epidemiology and Preventive Medicine, Monash University, Melbourne, Australia Medical Research Institute of New Zealand, Wellington, New Zealand rparke@adhb.govt.nz.

2Cardiothoracic and Vascular Intensive Care Unit, Auckland City Hospital, Auckland, New Zealand Australian and New Zealand Intensive Care Research Centre, Department of Epidemiology and Preventive Medicine, Monash University, Melbourne, Australia Medical Research Institute of New Zealand, Wellington, New Zealand.

3Cardiothoracic and Vascular Intensive Care Unit, Auckland City Hospital, Auckland, New Zealand.

Abstract

BACKGROUND:

After cardiac surgery, patients receive large amounts of fluid in the Intensive Care Unit (ICU). We plan to conduct a multi-centre randomised controlled trial, of a conservative fluid regime, in patients after cardiac surgery, and have reported results of a feasibility study that evaluated efficacy and safety of the proposed regime.

METHODS:

After ethical approval, a single-centre, prospectively randomised interventional study was undertaken. Participants were randomised to either usual care, or to a protocolised algorithm, utilising stroke volume variation, to guide fluid administration to patients who were deemed to have inadequate cardiac output and were likely to be volume responsive. The study protocol lasted from ICU admission to de-sedation or 24 h, whichever occurred first.

RESULTS:

We randomised 144 subjects over 9 months. Less bolus fluid and less total overall fluid volume was administered in the intervention group (median (IQR) 1620 ml (500-3410) and 2525 ml (1440-5250; P<0.001), compared with the usual care group (2050 ml (910-4280) and 2980 ml (2070-6580; P=0.001), from ICU admission to extubation. There was no significant difference in incidence of acute kidney injury or the average amount of fluid administered to the usual care group at the beginning compared with the end of the study.

CONCLUSION:

It is both possible and safe to achieve a significant reduction in the amount of fluid administered to patients, allocated to a conservative fluid protocol. These results suggest that a planned multi-centre study is both justified and feasible.

CLINICAL TRIAL REGISTRATION:

Australia New Zealand Clinical Trials Registry www.anzctr.org.au (ACTRN12612000754842).

© The Author 2015. Published by Oxford University Press on behalf of the British Journal of Anaesthesia. All rights reserved. For Permissions, please email: journals.permissions@oup.com.

KEYWORDS:

acute kidney injury; cardiac output; cardiac surgery; haemodynamics; intensive care units

PMID: 25991758

**3. Arch Dis Child 2014;99:A257 doi:10.1136/archdischild-2014-307384.699**

Adolescent Health

PO-0019 Non-invasive Haemodynamic Assessment Of Anaesthetic Induction In Adolescents – A Pilot Study

B Saikia1, H Wellesley2, J Brierley1

1PICU, Great Ormond St Children’s Hospital, London, UK

2Anaesthetic Department, Great Ormond St Children’s Hospital, London, UK

Abstract

Background Little is known about the haemodynamic effects of commonly used paediatric anaesthetic drugs in teenagers. Previous ‘gold-standard’ invasive haemodynamic monitoring is inappropriate in well children, whereas children undergoing haemodynamic monitoring on ICU are not drug-naive.

Aim 6-month study to explore initial haemodynamic effects of commonly used induction agents during elective anaesthesia for orthopaedic conditions in healthy stable children.

Method Consent from those with parental responsibility obtained. All haemodynamic assessment performed using a non-invasive Doppler ultrasound (USCOM). Data collected: stroke volume (SV), stroke volume index (SVI), cardiac output (CO), cardiac index (CI), stroke volume variability (SVV), heart rate (HR) and blood pressure (BP). Times: Immediately prior to induction, then at 10, 20 and 30 min post induction.

Results 9 patients studied – five female; age 11–15 years. Induction agents: Propofol 7; sevoflurane 1; nitrous 1. Descriptive hemodynamics: Propofol – slow and steady fall observed in SV, SVI, CO, CI, SBP, DBP and HR over time. Sevofluorane – same but BP drop more rapid. Nitrous – steady SV, SVI, CO and CI with slow fall in HR but more pronounced fall in BP. No definite pattern could be predicted for SVV.

Conclusion Whilst this data is clearly limited in terms of patient number and variety of agents, it is the first description of the haemodynamic effects of induction of anaesthesia in well children. Whilst there were no deleterious effects, an understanding of the haemodynamic effects of these commonly used agents might lead to safer anaesthesia in more complex and critically ill children.

**4. Korean J Anesthesiol. 2014 May;66(5):358-63. doi: 10.4097/kjae.2014.66.5.358. Epub 2014 May 26.**

The influence of different mechanical ventilator settings of peak inspiratory pressure on stroke volume variation in pediatric cardiac surgery patients.

Kang WS1, Kim JY2, Woo NS1, Yoon TG1.

Author information

1Department of Anesthesiology and Pain Medicine, Konkuk University Hospital, Konkuk University Medical Center, Konkuk University School of Medicine, Seoul, Korea. ; Research Institute of Medical Science, Konkuk University School of Medicine, Seoul, Korea.

2Department of Anesthesiology and Pain Medicine, Konkuk University Hospital, Konkuk University Medical Center, Konkuk University School of Medicine, Seoul, Korea.

Abstract

BACKGROUND:

The usefulness of dynamic parameters derived by heart-lung interaction for fluid responsiveness in pediatric patients has been revealed. However, the effects of peak inspiratory pressure (PIP) that could affect the absolute values and the accuracy in pediatric patients have not been well established.

METHODS:

Participants were 30 pediatric patients who underwent ventricular septal defect repair. After completion of surgical procedure and sternum closure, mean arterial blood pressure, heart rate, central venous pressure, cardiac output, cardiac index and stroke volume variation (SVV) were measured at PIP 10 cmH2O (PIP10), at PIP 15 cmH2O (PIP15), at PIP 20 cmH2O (PIP20) and at PIP 25 cmH2O (PIP25).

RESULTS:

SVV at PIP15 was larger than that at PIP10 (13.7 ± 2.9% at PIP10 vs 14.7 ± 2.5% at PIP15, P < 0.001) and SVV at PIP20 was larger than that at PIP10 and PIP15 (13.7 ± 2.9% at PIP10 vs 15.4 ± 2.5% at PIP20, P < 0.001; 14.7 ± 2.5% at PIP15 vs 15.4 ± 2.5% at PIP20, P < 0.001) and SVV at PIP25 was larger than that at PIP10 and PIP15 and PIP20 (13.7 ± 2.9% at PIP10 vs 17.4 ± 2.4% at PIP25, P < 0.001; 14.7 ± 2.5% at PIP15 vs 17.4 ± 2.4% at PIP25, P < 0.001; 15.4 ± 2.5% at PIP20 vs 17.4 ± 2.4% at PIP25, P < 0.001).

CONCLUSIONS:

SVV is affected by different levels of PIP in same patient and under same volume status. This finding must be taken into consideration when SVV is used to predict fluid responsiveness in mechanically ventilated pediatric patients.

KEYWORDS:

Cardiac surgical procedures; Fluid therapy; Ventilation

PMID: 24910727

**5. Br J Anaesth. 2013 Apr;110(4):586-91. doi: 10.1093/bja/aes467. Epub 2012 Dec 18.**

Prediction of fluid responsiveness in mechanically ventilated children undergoing neurosurgery.

Byon HJ1, Lim CW, Lee JH, Park YH, Kim HS, Kim CS, Kim JT.

Author information

1Department of Anesthesiology and Pain Medicine, Seoul National University Hospital, Seoul, Republic of Korea.

Abstract

BACKGROUND:

The purpose of this study was to evaluate the clinical usefulness of static and dynamic variables for the prediction of fluid responsiveness in children under general anaesthesia.

METHODS:

Thirty-three mechanically ventilated children received 10 ml kg(-1) colloid for 10 min while stable during surgery. Arterial pressure, heart rate, central venous pressure (CVP), and pleth variability index (PVI), in addition to variation in systolic pressure, pulse pressure (including Δdown and Δup), respiratory aortic blood flow velocity (ΔVpeak), and inferior vena cava diameter were measured before and after volume expansion. Patients were classified as responders to fluid loading if their stroke volume index (SVI) increased by at least 10%.

RESULTS:

There were 15 volume responders and 18 non-responders. Of the variables examined, ΔVpeak (r=0.516, P=0.004) and PVI (r=0.49, P=0.004) before volume expansion were significantly correlated with changes in SVI. The receiver-operating characteristic (ROC) curve analysis showed that PVI and ΔVpeak predicted fluid responsiveness. Areas under the ROC curves of PVI and ΔVpeak were statistically larger than that of CVP (P=0.006 and 0.014, respectively). However, those of other variables were similar to that of CVP.

CONCLUSIONS:

ΔVpeak and PVI can be used to predict fluid responsiveness in mechanically ventilated children under general anaesthesia. The other static and dynamic variables assessed in this study were not found to predict fluid responsiveness significantly in children.

CLINICAL TRIAL REGISTRATION:

ClinicalTrials.gov, NCT01364103.

PMID: 23250892

**6. Anaesthesia. 2011 Jul;66(7):582-9. doi: 10.1111/j.1365-2044.2011.06715.x. Epub 2011 May 3.**

Non-invasive prediction of fluid responsiveness in infants using pleth variability index.

Renner J1, Broch O, Gruenewald M, Scheewe J, Francksen H, Jung O, Steinfath M, Bein B.

Author information

1Department of Anaesthesiology and Intensive Care Medicine, University Hospital of Schleswig-Holstein, Campus Kiel, Germany. renner@anaesthesie.uni-kiel.de

Abstract

This prospective study assessed whether respiratory variations in velocity time integral, peak blood flow velocity and non-invasive pleth variability index are useful measurements in infants undergoing congenital heart surgery and determined threshold values that may help guide fluid administration. In 27 infants receiving mechanical ventilation, of mean (SD) weight 10.4 (6.3) kg, 13 increased their stroke volume index ≥ 15% following a fluid challenge and 14 did not. The best area under the receiver operating characteristic curve was for the echocardiographic-derived variables respiratory variation in blood peak flow velocity (area under the ROC curve = 0.92; p = 0.0002) and respiratory variation of the velocity time integral (area under the ROC curve = 0.84; p = 0.002). The pleth variability index also predicted fluid responsiveness (area under the ROC curve = 0.79; p = 0.01), in contrast to heart rate (area under the ROC curve = 0.53; p = 0.75) and central venous pressure (area under the ROC curve = 0.57; p = 0.52).

© 2011 The Authors. Anaesthesia © 2011 The Association of Anaesthetists of Great Britain and Ireland.

PMID: 21539529

**7. Impact of high frequency oscillatory ventilation on fluid responsiveness dynamic indices in a pae-diatric intensive care unit**

Girard, F. Maria, M. Le Tacon, S. Monin, P. 2011

F. Girard, Paediatric Intensive Care Unit, Brabois Children Hospital, Vandouvre-les- Nancy, France

Abstract

Objectives: High frequency oscillatory ventilation (HFOV) generates higher mean alveolar pressure (MalvP) than conventional mechanical ventilation (CMV) and abolishes breathing movements, creating a constant cardio-pulmonary interaction. We wanted to analyse the variation of cardiac index (CI) and stroke volume variation (SVV), which is a dynamic marker of fluid responsiveness, during HFOV and while switching back to CMV. Methods: In a prospective observational study, children who have needed invasive hemodynamic monitoring and HFOV for acute respiratory distress syndrome and were ready to switch to CMV were enrolled. MalvP, CI and SVV were noted at baseline during HFOV and 1, 5 and 10 minutes after the switch. Statistical analyse used the Friedman non-parametric test to compare paired samples, followed by the Mann-Whitney test for multiple paired comparisons. A p value < 0.05 was considered as significant. Results: By studying SVV over time in a single patient during HFOV, unexpected variations were found. 8 switches from VOHF to CMV were realised in 6 patients. Although there was a highly significant difference for MalvP between HFOV and CMV, regardless of the time after ventilator switch (p<0.0001), neither CI (p= 0.63) nor SVV (p=0.834) were significant. Conclusion: Switching to CMV does not improve CI. SVV is detectable and varies under HFOV, despite the absence of intrathoracic pressure cyclic changes. Switching from HFOV to CMV does not raise SVV, whereas its increase was expected, induced by reappearance of variable cardio-pulmonary interactions. These findings raise questions about the real meaning of fluid responsiveness dynamic indices.

**8. Predictive value of plethysmography variability index as a non invasive marker of fluid responsiveness in a paediatric intensive care unit**

Ferry, H. Girard, F. Le Tacon, S. Maria, M. Schweitzer, C. Monin, P. 2011

H. Ferry, Henri Poincare University, Vandoeuvre-les-Nancy, France

Abstract

Objectives: The prediction of fluid responsiveness is based on invasive monitoring, which is responsible for complications among critically ill children. We hypothesized that the Plethysmography Variability Index (PVI), a new, non-invasive marker, could safely assess fluid responsiveness in paediatric patients. Methods: We led a prospective, monocentric study in the Paediatric intensive care unit of the Nancy Children's Hospital. Children requiring invasive cardiac output monitoring were enrolled. Exclusion criteria were spontaneous breathing or cardiac arrhythmia. PVI was displayed by pulse oxymeters and compared every two hours to SVV, which is an invasive, validated fluid responsiveness marker obtained through pulse contour analysis. Correlation was tested using Spearman's method and a ROC curve was drawn to predict SW>10%. Ap < 0.05 was considered significant. Results: 14 patients aged from 26 days to 18 years old were enrolled. 5 of them were admitted for acute respiratory distress syndrome, 8 for shock and 1 for severe bleeding. 601 PVI-SVV couples were obtained. The correlation between PVI and SVV was significant (p<0.001), but weak, according to the Spearman determination coefficient (r2 = 0.112). The ROC curve showed an AUC of 0.654, significantly different from 0.5. The best threshold was 13% (Sensitivity 63%, Specificity 61%). Conclusion: PVI is correlated with SVV but cannot be used unfailingly to predict fluid responsiveness among critically ill children. This result differs from those obtained among adults. This can be explained by children's arterial properties, which include quick variations of tonus, compliance and reactance, to which SVV adjusts, unlike PVI.

**9. Pediatr Cardiol. 2010 Nov;31(8):1166-70. doi: 10.1007/s00246-010-9776-8. Epub 2010 Aug 13.**

Respiratory variation in aortic blood flow velocity as a predictor of fluid responsiveness in children after repair of ventricular septal defect.

Choi DY1, Kwak HJ, Park HY, Kim YB, Choi CH, Lee JY.

Author information

1Division of Cardiology, Department of Pediatrics, Gachon University of Medicine and Science, Incheon, 405-760, Republic of Korea.

Abstract

This study aimed to compare respiratory variation in transthoracic echo-derived aortic blood flow velocity (∆Vpeak) and inferior vena cava diameter (∆IVCD) with central venous pressure (CVP) as predictors of fluid responsiveness in children after repair of ventricular septal defect (VSD). A prospective study conducted in pediatric intensive care unit investigated 21 mechanically ventilated children who had undergone repair of VSD. Standardized volume replacement (VR) was the intervention used. Hemodynamic measurements including CVP, heart rate, mean arterial pressure, transthoracic echo-derived stroke volume (SV), cardiac output, ∆Vpeak, and ∆IVCD were performed 1 h after patient arrival in the intensive care unit. Hemodynamic measurements were repeated 10 min after VR by an infusion of 6% hydroxyethyl starch 130/0.4 (10 ml/kg) over 20 min. The volume-induced increase in the SV was 15% or more in 11 patients (responders) and less than 15% in 10 patients (nonresponders). Before volume replacement, the ∆Vpeak (23.1 ± 5.7% vs. 14.0 ± 7.7%; p = 0.006) and ∆IVCD (26.5 ± 16.2% vs. 9.2 ± 9.1%; p = 0.008) was higher in the responders than in the nonresponders, whereas CVP did not significantly differ between the two groups. The prediction of fluid responsiveness was higher with the ΔVpeak, as shown by a receiver operating characteristic curve area of 0.83 (95% confidence interval [CI], 0.61-1.00; p = 0.01), a ΔIVCD of 0.85 (95% CI, 0.69-1.00; p = 0.01), and a CVP of 0.48 (95% CI, 0.22-0.73; nonsignificant difference). The ∆Vpeak and ∆IVCD measured by transthoracic echocardiography can predict the response of SV after volume expansion in mechanically ventilated children at completion of VSD repair.

PMID: 20703453

**10. Intensive Care Med. 2001 Jan;27(1):201-5.**

Are transoesophageal Doppler parameters a reliable guide to paediatric haemodynamic status and fluid management?

Tibby SM1, Hatherill M, Durward A, Murdoch IA.

Author information

1Department of Paediatric Intensive Care, Guy's Hospital, London, UK.

Abstract

OBJECTIVE:

Transoesophageal Doppler (TOD) has been used in adults to optimise left ventricular filling on the basis of the waveform parameters. We wished to see if a similar relationship exists in children, specifically: (a) whether change in thermodilution stroke volume (SV) following a fluid bolus corresponded to change in Doppler stroke distance, Doppler corrected flow time (FTc), or central venous pressure (CVP); (b) whether a response to fluid challenge (defined as an increase in SV of greater than 10%) can be predicted on the basis of an absolute value for FTc or CVP prior to fluid bolus; and (c) the relationship between FTc and systemic vascular resistance index.

DESIGN:

Prospective, comparison study.

SETTING:

Sixteen-bed paediatric intensive care unit of a university hospital.

PATIENTS:

Ninety-four ventilated children were studied, median (range) age 25 months (4 days- 16 years). Diagnoses included: post-cardiac surgery (n = 58), sepsis/multi-organ failure (n = 29), respiratory disease (n = 5), and other (n = 2).

INTERVENTIONS:

A 4-MHz, 5.5-mm diameter, flexible TOD probe was placed when patients were haemodynamically stable. Five consecutive measurements of stroke distance and FTc were made and averaged, concurrently with five SV measurements by femoral artery thermodilution. SV was then augmented by administration of fluid (10 ml/kg), and haemodynamic recordings were repeated.

MEASUREMENTS AND MAIN RESULTS:

The median (range) SV was 17 ml (2-64 ml). The median coefficients of variation were 3.9 % for SV, 3.5 % for stroke distance, and 3.1% for FTc. Changes in SV were accurately tracked by changes in stroke distance (mean bias 1.8 %, limits of agreement +/- 17%), but not by FTc or CVP. FTc was weakly inversely correlated with systemic vascular resistance (r = -0.15, P < 0.05). Among non-cardiac patients (n = 36), the optimal FTc that predicted an improvement in SV following fluid bolus was 0.394 s (area under ROC curve 0.756), giving a sensitivity of 90 %, specificity of 62 %, positive predictive value of 47 %, and a negative predictive value of 94 %. CVP was a poor predictor for all patient groups.

CONCLUSIONS:

TOD stroke distance is able to follow changes in SV following fluid bolus amongst ventilated children, and can predict when further volume loading is unlikely to improve SV amongst general, but not cardiac ICU patients. CVP is a poor discriminator of volume status in this group of patients.

PMID: 11280635

**2 articles with patients enrolled in other included studies were excluded:**

**1. Crit Care. 2013; 17(Suppl 2): P207.**

Published online 2013 Mar 19. doi: 10.1186/cc12145

PMCID: PMC3642550

Prediction of stroke volume response to fluid bolus in 100 children

R Saxena,corresponding author1 A Durward,1 I Murdoch,1 and S Tibby1

Introduction

Fluid overload is associated with poor outcome in the critically ill. Thus, an accurate predictor of a positive haemodynamic response (increase in stroke volume) to fluid challenge is vital.

Methods

We studied the predictive value (positive response defined as change in stroke volume >15% after 10 ml/kg fluid bolus) of a range of haemodynamic variables: static (CVP, active circulating volume, central blood volume, total end diastolic volume), dynamic (systolic pressure variation, stroke volume variation) and contactility (dp/dt), in a group of 100 ventilated children (median weight 10 kg). Variables were measured using transpulmonary ultrasound dilution and PRAM (an arterial pulse contour method).

Results

We performed 168 paired measurements (pre-fluid and post-fluid challenge), with a SV response rate of 45%. Overall predictive values were poor, but slightly better for static versus dynamic variables (Table ​(Table1).1). When SV response was analysed as a continuous variable, the two predictive multivariable variables were change in TEDVI and baseline dp/dt (r2 = 0.30, both P <0.001).

Conclusion

The predictive ability for typical static and dynamic haemodynamic variables, when taken in isolation, is poor. However, improved prediction is seen when baseline contractility is taken into account.

**2. European Journal of Anaesthesiology: June 2013 - Volume 30 - Issue - p 77–77**

Prediction of fluid responsiveness by non‐invasive cardiac output monitoring and transthoracic echo in children after repair of ventricular septal defect: 4AP8‐2

Chang, Y. J.; Cho, Y. Y.; Kim, H. S.; Lee, J. Y.

Gachon Universit y Gil Medical Center, Dept of Anaesthesiology & Pain Medicine, Inchon, Korea, Republic of

Background and Goal of Study: Non-invasive cardiac output monitoring is a potentially useful clinical tool in the pediatric setting. This study aimed to compare stroke volume variation (SVV) in non-invasive cardiac output monitoring (NICOM) with respiratory variation in transthoracic echo-derived aortic blood flow velocity (ΔVpeak) as predictors of fluid responsiveness in children af ter repair of ventricular septal defect (VSD).

Materials and Methods: A prospective study conducted in pediatric intensive care unit investigated 26 mechanically ventilated chidlren who had undergone repair of VSD. Standardized volume replacement (VR) was the intervention used. Hemodynamic measurements including central venous pressure, heart rate, mean arterial pressure, transthoracic echo derived stroke volume (SV), cardiac output, ΔVpeak, and SVV in NICOM were performed 30 min after patient arrival in the intensive care unit. Hemodynamic measurements were repeated 10 min af ter VR by an influsion of 6% hydroxyethyl starch 130/0.4 (10 ml/kg) over 20 min.

Results and Discussion: The volume induced increase in the SV was 15% or more in 13 patients (responders) and less than 15% in 13 patients (nonresponders). Before volume replacement, the ΔVpeak (19 ± 6% vs. 9 ± 4%; p < 0.001) and SVV (13 ± 3% vs. 8 ± 2%; p < 0.001) was higher in the responders than in the non-responders. The prediction of fluid responsiveness was higher with the ΔVpeak, as shown by a receive operating characteristic curve area of 0.956 (95% confience interval [CI], 0.885 - 1.00; p = 0.001), a SVV of 0.888 (95% CI, 0.764 - 1.00; p = 0.001).

Conclusion(s): The ΔVpeak and SVV can predict the response of SV after volume expansion in mechanically ventilated children at completion of VSD repair.

**2 articles with incomplete original data were excluded:**

**1. Pediatr Crit Care Med. 2015 Mar;16(3):e89-94.**

doi: 10.1097/PCC.0000000000000364.

Low predictability of three different noninvasive methods to determine fluid responsiveness in critically ill children.

Weber T1, Wagner T, Neumann K, Deusch E.

Author information

11Department of Anesthesia and Critical Care, Danube Hospital, KAV-Vienna, Vienna, Austria. 2Department of Pediatrics, Pediatric Intensive Care Unit, Danube Hospital, KAV-Vienna, Vienna, Austria. 3Department of Biometry and Clinical Epidemiology, Campus Charité Benjamin Franklin, Charité-Medicine University Berlin, Berlin, Germany. 4Department of Anesthesia and Critical Care, Hanuschkrankenhaus, WGKK, Vienna, Austria.

Abstract

OBJECTIVE:

To predict fluid responsiveness by noninvasive methods in a pediatric critical care population.

DESIGN:

Prospective observational clinical trial.

SETTING:

PICU in a tertiary care academic hospital.

PATIENTS:

Thirty-one pediatric patients aged from 1 day to 13 years under mechanical ventilation and on catecholamine support.

INTERVENTIONS:

We tested three noninvasive methods to predict fluid responsiveness: an esophageal Doppler system (CardioQ), a pulse contour analysis algorithm system (LiDCOrapid), and respiratory variations in vena cava inferior diameter. Stroke volume index was measured by transthoracic echocardiography before and after fluid challenge to determine fluid responders. Infusion of 10 mL/kg hydroxyethylstarch 130/0.4.

MEASUREMENTS AND MAIN RESULTS:

Predictability of fluid responsiveness was only found in Doppler peak velocity of descending aortal blood flow. Increased peak velocity with reduction after fluid bolus was predictive for nonresponding to IV fluid challenge. Sensitivity and specificity of peak velocity were 69% and 73%, respectively. The cut point was set at 135.5 cm/s. The lower the Doppler peak velocity, the higher was the probability for a fluid response. Neither stroke volume variations nor respiratory variations in vena cava inferior diameter during mechanical ventilation were useful in predicting fluid responsiveness in this pediatric patient population. None of the children had abdominal hypertension measured by bladder pressure.

CONCLUSIONS:

Dynamic preload variables such as stroke volume variation or respiratory variations in vena cava inferior diameter may not be useful for predicting fluid responsiveness in certain pediatric patient populations. Esophageal Doppler peak velocity was predictive of fluid responsiveness where a target value of more than 135,5 cm/s may be a signal to terminate further fluid challenges. This target value may be different in different age groups, as esophageal Doppler peak velocity varies with age.

PMID: 25647238

**2. J Clin Monit Comput. 2007 Feb;21(1):1-6. Epub 2006 Nov 11.**

Variation in blood pressure as a guide to volume loading in children following cardiopulmonary bypass.

Tran H1, Froese N, Dumont G, Lim J, Ansermino JM.

Author information

1Department of Electrical & Computer Engineering, The University of British Columbia, Vancouver, V6H 3V4, Canada.

Abstract

OBJECTIVE:

Intravascular volume loading is used to optimize cardiac output in children following weaning from cardiopulmonary bypass. Central venous pressure (CVP) is frequently used to titrate fluid administration but it is often misleading in predicting fluid responsiveness. Variation in the arterial pressure waveform is exaggerated in patients with deficient intravascular volume and has been shown to be a good predictor of fluid responsiveness in adults following cardiac surgery. The aim of this study was to compare the measures of variation in blood pressure as a guide to volume loading in children following cardiopulmonary bypass.

METHODS:

After ethical approval, we collected continuous real-time measurements from 25 children during volume loading after cardiopulmonary bypass. Subjects with moderate or severe tricuspid incompetence or who did not require volume loading during weaning from cardiopulmonary bypass were excluded from the study. Unstable readings were excluded from analysis. Systolic Pressure Variation (SPV), Pulse Pressure Variation (PPV) and Systolic Volume Variation (SVV) were retrospectively calculated before and after each bolus of fluid. Fluid responsiveness was classified as a change in blood pressure of > or =80 mmHg/L/m(2).

RESULTS:

Forty-four boluses were analyzed from the 25 children. Respiratory variables were similar. CVP was a poor predictor of fluid responsiveness and a negative relationship between change in blood pressure and Delta Down was observed. Performance in predicting fluid responsiveness as measured by the areas under the ROC curves were CVP (0.58), PPV (0.67), SPV (0.74) and SVV (0.74).

CONCLUSIONS:

Variation in blood pressure was a better guide to volume loading in children than CVP. Delta down was not useful in predicting fluid responsiveness in children with open chests following bypass surgery. SPV and SVV require further testing in prospective clinical trials.

PMID: 17103015

**2 articles review were excluded:**

**1. Anesth Analg. 2013 Dec;117(6):1380-92. doi: 10.1213/ANE.0b013e3182a9557e.**

Predicting fluid responsiveness in children: a systematic review.

Gan H1, Cannesson M, Chandler JR, Ansermino JM.

Author information

1From the *Department of Anesthesiology, Pharmacology, and Therapeutics, University of British Columbia; †Department of Anesthesia, BC Children's Hospital, Vancouver, Canada; ‡Department of Anesthesiology and Perioperative Care, University of California, Irvine, School of Medicine, Irvine, California; and §Department of Anaesthesia, University College London Trust, London, United Kingdom.

Abstract

BACKGROUND:

Administration of fluid to improve cardiac output is the mainstay of hemodynamic resuscitation. Not all patients respond to fluid therapy, and excessive fluid administration is harmful. Predicting fluid responsiveness can be challenging, particularly in children. Numerous hemodynamic variables have been proposed as predictors of fluid responsiveness. Dynamic variables based on the heart-lung interaction appear to be excellent predictors of fluid responsiveness in adults, but there is no consensus on their usefulness in children.

METHODS:

We systematically reviewed the current evidence for predictors of fluid responsiveness in children. A systematic search was performed using PubMed (1947-2013) and EMBASE (1974-2013). Search terms included fluid, volume, response, respond, challenge, bolus, load, predict, and guide. Results were limited to studies involving pediatric subjects (infant, child, and adolescent). Extraction of data was performed independently by 2 authors using predefined data fields, including study quality indicators. Any variable with an area under the receiver operating characteristic curve that was significantly above 0.5 was considered predictive.

RESULTS:

Twelve studies involving 501 fluid boluses in 438 pediatric patients (age range 1 day to 17.8 years) were included. Twenty-four variables were investigated. The only variable shown in multiple studies to be predictive was respiratory variation in aortic blood flow peak velocity (5 studies). Stroke volume index, stroke distance variation, and change in cardiac index (and stroke volume) induced by passive leg raising were found to be predictive in single studies only. Static variables based on heart rate, systolic arterial blood pressure, preload (central venous pressure, pulmonary artery occlusion pressure), thermodilution (global end diastolic volume index), ultrasound dilution (active circulation volume, central blood volume, total end diastolic volume, total ejection fraction), echocardiography (left ventricular end diastolic area), and Doppler (stroke volume index, corrected flow time) did not predict fluid responsiveness in children. Dynamic variables based on arterial blood pressure (systolic pressure variation, pulse pressure variation and stroke volume variation, difference between maximal or minimal systolic arterial blood pressure and systolic pressure at end-expiratory pause) and plethysmography (pulse oximeter plethysmograph amplitude variation) were also not predictive. There were contradicting results for plethymograph variation index and inferior vena cava diameter variation.

CONCLUSIONS:

Respiratory variation in aortic blood flow peak velocity was the only variable shown to predict fluid responsiveness in children. Static variables did not predict fluid responsiveness in children, which was consistent with evidence in adults. Dynamic variables based on arterial blood pressure did not predict fluid responsiveness in children, but the evidence for dynamic variables based on plethysmography was inconclusive.

PMID: 24257389

**2. Fluid Therapy Should be Guided by Fluid Responsiveness**

Article (PDF Available) in Archives of Disease in Childhood 97(Suppl 2):A3-A4 · October 2012 with 12 Reads

DOI: 10.1136/archdischild-2012-302724.0011

Jesús López-Herce,Javier Urbano

Institution

Hospital General Universitario Gregorio Marañón

Department

Pediatric Intensive Care Unit

Abstract

Background To predict fluid response is very Important because a little or excessive expansion may alter the prognosis of the child in shock. Methods We review experimental and clinical articles in adult and children about parameters that could predict fluid responsiveness in shock. We also analyze our experimental data in pediatric experimental model of hemorrhagic shock. Results The most used parameters to try to predict hemodynamic response to fluids are: static pressure parameters as central venous pressure (CVP); volume as global end diastolic ventricular index (GEDVI) or stroke volume index (SVI); dynamic parameters, as pulse pressure variation (PPV) and systolic volume variation (SVV), and the response to a maneuver that increases blood volume without expanding the patient (leg raises). Several studies in adults suggest that hemodynamic volume parameters (SVI or GEDVI) predict better the response to fluids than pressure parameters (PVC); that dynamic parameters (PPV and SVV) predict better the response to fluids that static parameters; and that maneuver leg raises maneuver is the best predictive parameter. However, the results of other studies are contradictory. In children there are few studies and there is no evidence that dynamic parameters are better predictors than static volume parameters. Our experimental studies confirm these findings. Preliminary data suggest that leg raise maneuver has not good predictive power in children. Conclusion at this time fluid therapy in children with shock should be guided by fluid responsiveness. Macrohemodynamic, microhemodynamic and tissue parameters should be used to control the response to fluid therapy.
