# Supplementary figures and images for: Does stroke volume variation predict fluid responsiveness in children: A systematic review and meta-analysis
Source: PLoS One. 2017 May 12;12(5):e0177590. doi: 10.1371/journal.pone.0177590 (PMC5428964; doi:10.1371/journal.pone.0177590)

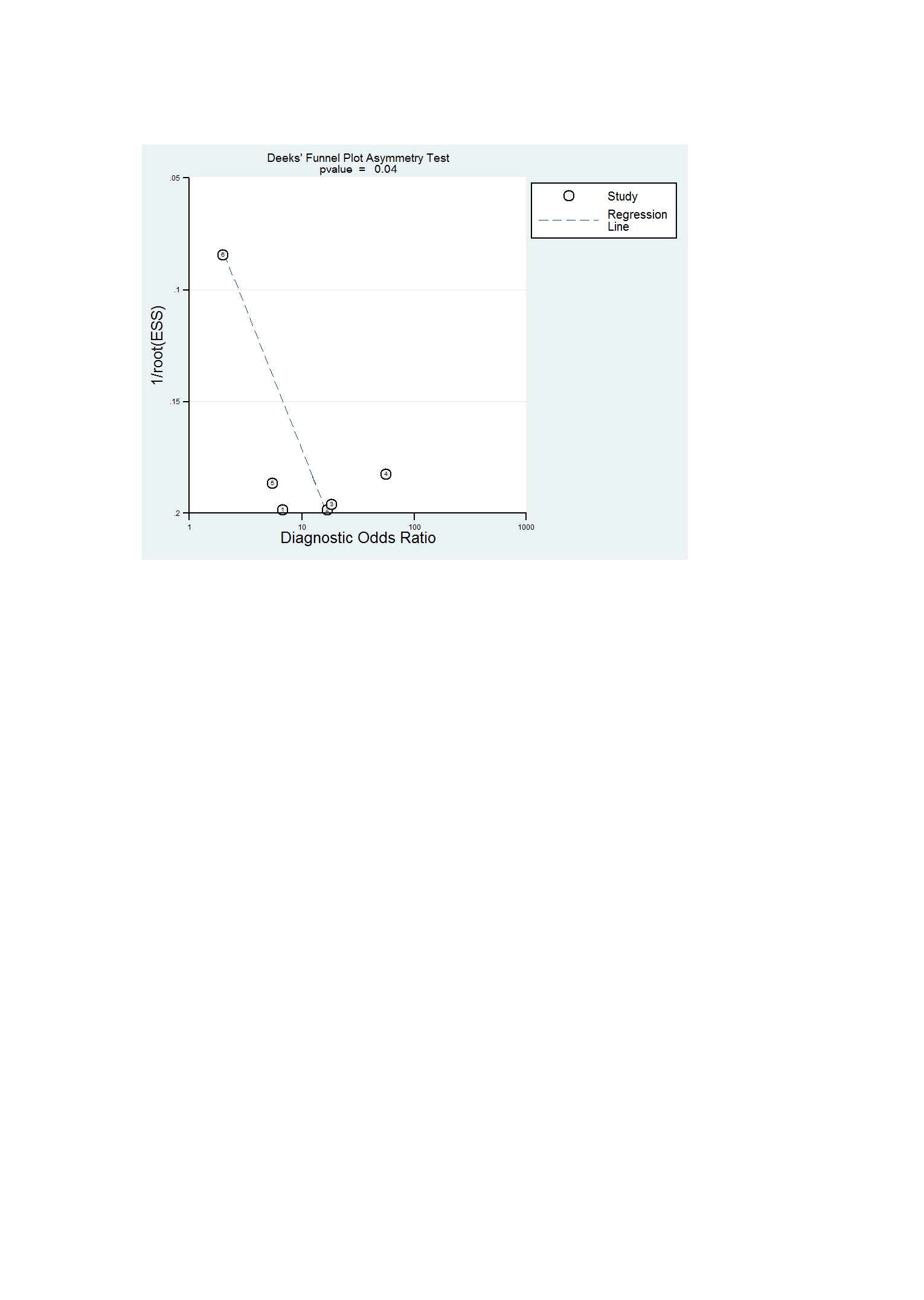

Supplement: S1 Fig — (TIFF) [file pone.0177590.s003.tiff]
